# Social network tie functions of social support and social influence and adult smoking abstinence

**DOI:** 10.1371/journal.pone.0296458

**Published:** 2024-03-07

**Authors:** Cynthia M. Lakon, Yu Zheng, Cornelia Pechmann

**Affiliations:** 1 Health, Society, & Behavior, Program in Public Health, University of California, Irvine, Irvine, California, United States of America; 2 School of School of Journalism and Communication, Sun Yat-sen University, Panyu District, Guangzhou, P.R. China; 3 Paul Merage School of Business, University of California Irvine, Irvine, Irvine, California, United States of America; Indiana University School of Medicine, UNITED STATES

## Abstract

Adults’ social network ties serve multiple functions and play prominently in quitting smoking. We examined three types of adults’ egocentric social networks, including family, friends, and friends online to investigate how two network characteristics with major relevance to health behavior, network size and tie closeness, related to the emotional and confidant support and to pro- and anti-smoking social influence these ties may transmit. We also examine whether the social support and social influence constructs related to smoking abstinence. We utilized baseline and 7-day abstinence survey data from 123 adult current smokers attempting to quit prior to the start of a randomized controlled quit-smoking trial of a social support intervention for quitting smoking on Twitter. To examine study relationships, we estimated Negative Binomial Regression models and Logistic Regression models. For all networks, network size and tie closeness related positively to most of the social support and social influence constructs, with tie closeness related most strongly, especially for online friends. Family pro-smoking social influence related negatively to smoking abstinence, and there were marginally negative relationships for family emotional support and family confidant support. Online friend emotional support had a marginally positive relationship with smoking abstinence. Overall, our findings indicated the importance of the social support and social influence functions of each type of network tie, with larger networks and closer ties related to higher levels of social support and social influence. Moreover, family network pro-smoking social influence may compromise abstinence while emotional support from online friend network ties may reinforce it.

## Introduction

Studies over decades indicate that social network ties impact health and health behavior [1–5]. This study focuses on three major types of social network ties adults maintain that are influential for health behavior, including family, friends, and friends online. While studies indicate the importance of family and friend ties for smoking and smoking cessation among adults [6, 7], studies have also begun to demonstrate the importance of online ties for health behavior [8, 9]. Social media including Twitter are portals to online social worlds, representing novel opportunities for creating and maintaining social relationships. Approximately 70% of Americans use social media to connect with one another and share information [10]. Yet, whether and how online social network ties influence health behavior, and what kinds of social support and social influences for health these online ties provide, have not been comprehensively studied.

### Adult egocentric network characteristics and health behavior

Herein, we examined adults’ egocentric networks of family, friend, and online friend ties which are defined from the vantage point of a focal individual and include their proximal social ties defined for some role relationship [11]. We focus on two network characteristics with major relevance for health behavior, including the number of network ties adults maintain, namely network size, and the closeness of these ties. Network size, when defined egocentrically, indicates the number of people an individual names in a network defined for some role relationship. Research indicates that both network size and the closeness of network ties inversely relate to mortality risk [1, 12]. Network size has been positively associated with beneficial health outcomes [13, 14], and consistently and negatively associated with risk behaviors [5, 15], with some exceptions to this pattern [16].

The closeness of network ties also has major relevance for health behavior, as individuals tend to engage in both risk-promotive and risk-protective behaviors with their close ties [16, 17]. Closeness is one dimension of tie strength [18, 19]. Stronger ties have the potential to be more supportive than weaker ties [20], as one study indicated that tie strength positively related to the provision of emotional support, companionship, and services [21]. Strong ties, however, may be supportive in certain instances only rather than more generally [21]. Tie strength may also impact the amount of social influence ties can transmit, with stronger ties being more influential than weak ties [22].

### Adult egocentric networks and social support

We study how the two network characteristics, network size and closeness of ties, relate to theoretically salient domains of social support for health behavior–emotional support [4] and confidant support [23, 24]—across the three networks. One of the primary functions of social network ties for health is the provision of social support [4, 25–27]. Emotional support encompasses feelings of encouragement, closeness, connection, and belongingness [4]. Emotional support has been most clearly related to health through both direct and buffering effects [28], and yet it remains one of the most elusive domains of social support for health because it relates to both salutary health outcomes [28] and adverse health outcomes [27, 29, 30]. Emotional support can be detrimental to health via support for behaviors which are detrimental to health [16].

The second domain of social support under study is confidant support, which encompasses feelings of deep closeness and the sharing of inner-most thoughts and feelings with another [23, 24]. Confidant support has been related to health outcomes in numerous studies [31–33]. Confidant support from online ties merits study as adults might self-disclose in ways that are different with friends online than with those in-person, given that a disinhibition effect can occur when communicating online [34].

The social support transmitted via network ties is related to both network size and tie closeness. Studies indicate that larger networks can provide more emotional support [35, 36] in comparison to smaller networks [37]. Findings regarding the relationship between the closeness of network ties and social support are mixed; close ties can in some instances increase access to emotional support resources, however may simultaneously limit access to social resources outside of a network [38].

Family members are a primary source of social support for adults [39], specifically emotional support [40]. Support from family members has been associated with emotional wellbeing [41]. Friends are another important source of social support for adults. Young adults tend to have more friends than family in their support networks, whereas older adults have fewer friends relative to family members [42].

Social support from family and friends influences health by affecting the performance of health-related behaviors including quitting smoking. One study found that social support from spouses or intimate partners, other family members, and friends related to reductions in smoking, but was less effective in helping women in comparison to men [6]. Other research found that increasing family members’ capacity to provide support for quit attempts was useful in helping smokers quit [43]. Moreover, other work showed that social support for quitting, measured as the amount of support the smoker received from co-workers, friends, family, supervisors and employers, was associated with stronger intentions to quit smoking and perceived self-efficacy [7]. It is possible that the effects of social support from family and friends may not always be helpful for quitting smoking, and that sometimes these ties may act as conduits of pro-smoking influence which can encourage continued smoking or exert other kinds of social influences which are counterproductive for quitting.

A third and increasingly relevant dimension of adults’ social worlds is their friends online. Online platforms may facilitate friendship formation among adults, whose lives can be characterized by multiple and complex daily roles and limited time for friendships. Moreover, online ties may not carry the same relational demands which are required to maintain in-person ties. However, much is still unknown about how adults perceive these online ties, specifically whether or not they perceive online ties as sources of social support, or as circumscribing their social networks and encouraging social isolation [44]. Moreover, if these ties are perceived as supportive by adults, what kinds of social support are they perceived to confer? Studies indicate that online social platforms offer access to a diverse set of people who may confer various types of social support, including emotional and informational support [45, 46]. One study found that individuals using Twitter sought and gained informational support [47]. Other research indicates that having more online social ties and communicating frequently with those people related to more closeness and social support [48]. We are not aware of studies which have examined the relationship between egocentric network social support from friends online independent from those formed in a health behavior change intervention targeting adult smoking cessation.

### Adult egocentric networks and social influence

In addition to social support, a second major function of social network ties for health is social influence [25]. Researchers have long focused on the influence properties of social network ties [49], both health promotive and health compromising. Studies suggest that family and friends may be major conduits of social influence for adult smoking cessation behavior. One study found that having a spouse, sibling, or a friend who quit smoking was related to an increased chance of the respondent quitting [50]. In another study, increased reports of a spouse or partner exerting anti-smoking influence, or other family members or friends doing so, were each associated with greater decreases in men’s smoking; however, for women, these effects were weaker [6]. Hence, we study both pro-smoking and anti-smoking influences, to capture both the health compromising and the health enhancing properties of social influence transmitted through the network ties we examined.

Social influence has also been related to network size and tie closeness. Individuals are more likely to be influenced by their close social network ties than weak ties regarding health behaviors [51]. Moreover, larger networks may result in more social influence because there are more individuals who can exert and enforce conformity to these influences [52].

In the current study, we hypothesize that two theoretically salient characteristics of adults’ family, friend and online friend network ties—network size and the closeness of network ties–will relate to the social support and social influence constructs under study. Based on insights from existing literature, we predict generally positive relationships between both network size and tie closeness and the social support and social influence constructs under study provided by the friend and family network ties. Regarding the online friend network ties, we do not make predictions, because it is not yet clear from the literature how the social network characteristics of naturally occurring (i.e., outside of a health behavior change intervention) online friend ties relate to social support and social influence. In addition, we predict that emotional and confidant social support and anti-smoking social influence should relate positively to smoking abstinence, while pro-smoking social influence should relate negatively to smoking abstinence. We expect these relationships to be significant across both the family and friend networks, with those for the online friend network ties being less certain.

## Data and methods

### Data

We utilized survey data from 123 adult current smokers attempting to quit prior to the start of a randomized controlled quit-smoking trial. The data for this study came from a baseline survey and 7-day abstinence survey of a randomized controlled trial of a social support intervention for quitting smoking provided on Twitter. The survey assessed their family, friend, and online friend network ties, and their perceptions of the social support and social influence provided by each person they named. Their 7-day smoking abstinence was also assessed. The data collection and analysis methods in this study comply with the terms and conditions for the source of the data.

Participants were recruited from November 2011 through January 2014 via the Google search engine using Google AdWords. Study inclusion criteria were having smoked 100 or more cigarettes in their lifetime, smoking 5 or more cigarettes per day currently, being a frequent Facebook user, being prepared to quit in the next 30 days, being 18 to 59 years old, English speaking, living in the continental United States, having an active e-mail account, having a mobile phone with unlimited texting, and texting weekly. Exclusion criteria were contraindications to the nicotine patches which were provided as part of the intervention or taking a prescription medicine for depression or smoking cessation. The social network characteristics and the social support and social influence measures were collected approximately 2–4 weeks before the trial commenced. These data were collected from participants in both the treatment and control arms of the randomized controlled trial. Preliminary analyses showed consistent results for the social support, social influence, and 7-day abstinence measures for both sets of participants, and so the data were aggregated in the analyses reported here. Approval for the research was obtained from the Institutional Review Board at the university where the study was conducted.

### Measures

We elicited the three types of social networks egocentrically [11], as respondents were asked to name their closest family members, their friends, and their online friends. We constructed measures of the two social network characteristics, and the social support and social influence constructs, for each of the three types of network ties—family, friends, and online friends. We obtained a measure of 7-day smoking abstinence. We also measured demographic variables including age, gender, ethnicity, marital status, and education. In addition, we measured the number of cigarettes per day smoked. All variables were measured at baseline with the exclusion of the 7-day smoking abstinence measure.

#### Network size

We measured network size for the family, friend, and online friend networks. The question measuring *Family Network Size* was: “Name the people in your family you feel close to. Please list them by name, and only provide their first name and last initial or a nickname. You can name up to 50 people.” We coded every name a participant provided with a 1 and then summed these values to determine the family network size.

The question eliciting *Friend Network Size* was “Who are your friends? Please list them by name, and only provide their first name and last initial or a nickname. You can name up to 50 people.” We coded every name a participant provided with a 1 and then summed up these values to determine the friendship network size.

The question eliciting *Online Friend Network Size* was “Do you have people whom you consider to be your friends from Twitter, Facebook, MySpace or another social networking site you are on? If yes, please list anyone you consider to be a friend below, and only provide their first name and last initial or a nickname. You can name up to 50 people.” We coded every name a participant provided with a 1 and then summed up these values to determine the online friend network size.

#### Closeness of ties

We measured network tie closeness for the family, friend and online friend networks. *Family Network Tie Closeness* was measured using the question “How close do you feel to each person in your *family* network? Please choose one number to describe how close you feel to each person, using the following: 1 = not close at all, 2 = not very close, 3 = somewhat close, 4 = neither close nor not close, 5 = close, 6 = very close, 7 = extremely close.” If a respondent listed a family member but did not assign that person a closeness score, then such missing values were coded to 1 to avoid listwise deletion, and 0 was not a value of the variable. We summed the closeness scores for each family member the respondent reported on and then divided this sum by family network size.

*Friend Network Tie Closeness* was measured using the question “How close do you feel to each person in your *friendship* network?” using the same 1–7 scale as above, and the same missing value coding as above. We summed the closeness scores for each friend the respondent reported on and then divided this sum by friend network size.

*Online Friend Network Tie Closeness* was measured by asking: “How close do you feel to each person in your *online friend* network?” using the same 1–7 scale as above, and the same missing value coding as above. We summed the closeness scores for each online friend the respondent reported on and then divided this sum by online friend network size.

#### Emotional support

We measured two indicators of emotional support for the family, friend and online friend networks, respectively. For the family network, the first question was “Can you count on the people in your *family* network to listen to you when you need to talk?” The second question was “Can you count on the people in your *family* network to comfort you when you are very upset?” For both questions, participants were instructed to: “Please put a check mark on the line to indicate which person(s) below you can count on to listen to you when you need to talk. If none of the people named below will listen to you when you need to talk, please check the “none” box and move on to the next question.” We coded each person named in participants’ family networks either a 1 if they were perceived as providing this type of support or a 0 if not, for each of the two questions measuring emotional support. Emotional support for friends and online friends was measured similarly. We then summed the responses for each variable and summed the sums to create a composite measure for each network (family, friend, online friend). For each network, the two replicate items used to create the composite measure of emotional support were correlated (for family r = .89, for friends r = .89, for online friends r = .87).

#### Confidant support

We measured two indicators of confidant support for the family, friend, and online friend networks, respectively. For the family network, the first question was “Can you share your inner-most thoughts and feelings with the people in your *family* network?” The second question was “Can you be totally yourself around people in your *family* network?” For both indicators, respondents were asked to place a check mark on the line to indicate which person(s) provide this kind of support to them. We coded each person a participant named in their family network a 1 if the participant perceived that the person provided this support or a 0 if not, for each of these two questions. Confidant support of friends and online friends was measured similarly. We then summed the responses for each variable and then summed the sums to create a composite measure of confidant support for each network. For each network, the two replicate items used to create the composite measure of confidant support were correlated (for family r = .68, for friends r = .57, for online friends r = .62).

#### Anti-smoking social influence

We measured two indicators of anti-smoking social influence for the family, friend, and online friend networks, respectively. Regarding the family network, the first question was “Does anyone in your *family* network give you information about the risks of smoking?” The second question was “Does anyone in your *family* network give you information about quitting smoking?” For both indicators, we asked respondents to place a check mark on the line to indicate which person(s) provide this information. We coded each person a participant named in their family network either a 1 if the participant perceived that the person provided this type of information or a 0 if not. Anti-smoking social influence from friends and online friends was measured similarly. We then summed the responses for each variable and summed the sums to create a composite measure of anti-smoking social influence for each network. The two items used to create the anti-smoking social influence composite variable were correlated for each network (for family r = .70, for friends r = .67, for online friends r = .63.

#### Pro-smoking social influence

We included two indicators of pro-smoking social influence for the family, friendship, and online friend networks, respectively. Regarding the family network, the first question was “Do any of the people in your *family* network smoke cigarettes regularly?” The second question was “Does anyone in your *family* network think that smoking is acceptable?” We asked participants to place a check mark to indicate which person(s) smoked regularly, and which person(s) thought that smoking was acceptable. We coded each person named in their family network either a 1 if they were perceived to have provided this type of influence or a 0 if not, for each of these two indicators. Pro-smoking social influence from friends and online friends were measured similarly. We then summed the responses for each variable and summed the sums to create a composite measure of pro-smoking social influence for each network. The two items used to create the pro-smoking social influence composite variable were correlated for each network (for family r = .62, for friends r = .57, for online friends r = .81).

#### 7-day abstinence

Participants were required to specify a quit date that fell within 10 days of the start of the trial. We measured 7-day smoking abstinence based on each participant’s specified quit date by asking the participant “How many cigarettes have you smoked in the past 7 days?” and “Have you puffed on a cigarette within the past 7 days?” Any smoking or puffing was recorded as non-abstinent and coded as 0, while abstinence was coded as 1.

#### Demographics

The following demographic variables were included in all of our analyses. Age was measured continuously by asking: “How old are you? Provide numeric values only.” Gender was measured by asking: “What is your gender? (1 = male, 2 = female, 3 = transgender).” Ethnicity was measured using this question: “Are you Caucasian/white? (0 = no, 1 = yes).” Marital status was measured by asking: “What is your marital status? (0 = not married,1 = married).” Education was measured using the question: “Please indicate your education (check highest level completed) (1 = No formal education to 9 = A graduate degree).” In addition to these demographic variables, we also controlled for the number of cigarettes per day smoked, which was measured continuously.

#### Analyses

We first examined relationships between the network characteristics of size and closeness and the social support and social influence variables. Given that our social support and social influence dependent variables were count variables, we estimated Negative Binomial Regression models using STATA version 13. We regressed each social support and social influence variable, one at a time, in separate models onto both network characteristics, the demographics and number of cigarettes per day smoked in each model. The variance inflation factors (VIF) were less than 4 when both network characteristics were included in the same model and the correlation between the two network characteristics was less than r = .6 for all three networks. Therefore, we included both network characteristics in each model. The models were estimated for each type of network.

We next examined relationships between the social support and social influence variables and the 7-day abstinence outcome. Given the binary dependent variable, we used Logistic Regression models to regress the 7-day abstinence measure onto each social support measure and each social influence variable, separately, including demographic variables and number of cigarettes per day smoked in each model. These models were also estimated for each type of network.

Our final analytical sample consisted of 123 participants, based on a full sample of 154 participants but with 31 excluded including 27 participants who did not write down names for any of the three social networks and four participants did not respond to either abstinence question, and so they were excluded from the analytical sample, which is consistent with standard practice [53]. We utilized a power calculator [54] to determine the power of our sample size. We determined that for an odds ratio increase of 1.7 based on a one standard deviation change in an independent variable, the needed sample size is 110 to have power of .80, (i.e., 1 minus the Type II error of .20). This calculation was based on the size of the effect of the independent variable Family Network Pro-Smoking Social Influence. Our study sample size exceeds the 110 sample size needed as it is 123, and therefore has sufficient power for detecting this effect at .80.

## Results

In the final analytical sample (n = 123), participants’ mean age was 37. Most participants were female (74.8%), and the majority were Caucasian/White (89.4%). Moreover, 59.3% were married, and 46.3% had completed some college. Participants smoked 17.06 cigarettes per day on average. Fifty-one percent of participants reported 7-day smoking abstinence. Table 1 displays the participant descriptive statistics.

**Table 1:** Descriptive statistics: Demographics, cigarettes per day smoked, social network characteristics, social support, social influence and 7 day abstinence for the family, friend, and online friend networks (n = 123).

	Variable	Mean (SD)/Proportion
	Age	37.54(9.56)
	Gender	male:24.4%; female: 74.8%;transgender:0.8%
	Ethnicity	Caucasian/white: 89.4%
	Marital status	married: 59.3%
	Education	complete high school:26.0%; some college years completed:46.3%; complete college:20.3%; others:7.4%
	Cigarettes per day smoked	17.06(8.62)
	7-Day Abstinence	.51(.50)
**Family Network**	Family Network Size	4.21(3.33)
	Family Network Tie Closeness	5.86(1.41)
	Family Network Emotional Support	5.76(5.43)
	Family Network Confidant Support	5.37(4.53)
	Family Network Anti-Smoking Social Influence	2.79(2.80)
	Family Network Pro-Smoking Social Influence	2.18(2.36)
**Friend Network**	Friend Network Size	3.02(3.68)
	Friend Network Tie Closeness	4.56(2.25)
	Friend Network Emotional Support	4.33(5.34)
	Friend Network Confidant Support	3.93(4.77)
	Friend Network Anti-Smoking Social Influence	1.57(2.71)
	Friend Network Pro-Smoking Social Influence	1.92(2.85)
**Online Friend Network**	Online Friend Network Size	1.37(2.88)
	Online Friend Network Tie Closeness	2.48(2.17)
	Online Friend Network Emotional Support	1.45(2.76)
	Online Friend Network Confidant Support	1.50(3.12)
	Online Friend Network Anti-Smoking Social Influence	.50(1.21)
	Online Friend Network Pro-Smoking Social Influence	.75(1.91)

### Family network

*Family Network Size* related significantly and positively to *Family Network Emotional Support* (b = .15, p < .01), *Family Network Confidant Support* (b = .14, p < .01), *Family Network Anti-Smoking Social Influence* (b = .13, p < .01) and *Family Network Pro-Smoking Social Influence* (b = .16, p < .01). *Family Network Tie Closeness* related significantly and positively to *Family Network Emotional Support* (b = .14, p < .01), *Family Network Confidant Support* (b = .19, p < .01), and *Family Network Anti-Smoking Social Influence* (b = .20, p < .05), but not to *Family Network Pro-Smoking Social Influence* (b = -.01) (see Table 2).

**Table 2:** Negative Binomial Regression models of social support and social influence constructs regressed onto network characteristics and demographics, cigarettes per day smoked for family, friend and online friend networks (n = 123).

**Family Network Emotional Support**	**Β(SE)**
Family Network Size	.15(.01)***
Family Network Tie Closeness	.14(.04)***
**Family Network Confidant Support**	
Family Network Size	.14(.01)***
Family Network Tie Closeness	.19(.05)***
**Family Network Anti-Smoking Social Influence**	
Family Network Size	.13(.03)***
Family Network Tie Closeness	.20(.08)**
**Family Network Pro-Smoking Social Influence**	
Family Network Size	.16(.03)***
Family Network Tie Closeness	-.01(.08)
**Friend Network Emotional Support**	
Friend Network Size	.17(.02)***
Friend Network Tie Closeness	.36(.05)***
**Friend Network Confidant Support**	
Friend Network Size	.15(.01)***
Friend Network Tie Closeness	.37(.05)***
**Friend Network Anti-Smoking Social Influence**	
Friend Network Size	.16(.03)***
Friend Network Tie Closeness	.43(.09)***
**Friend Network Pro-Smoking Social Influence**	
Friend Network Size	.18(.03)***
Friend Network Tie Closeness	.22(.07)***
**Online Friend Network Emotional Support**	
Online Friend Network Size	.16(.05)***
Online Friend Network Tie Closeness	.73(.09)***
**Online Friend Network Confidant Support**	
Online Friend Network Size	.17(.04)***
Online Friend Network Tie Closeness	.61(.07)***
**Online Friend Network Anti-Smoking Social Influence**	
Online Friend Network Size	.09(.05)†
Online Friend Network Tie Closeness	.74(.13)***
**Online Friend Network Pro-Smoking Social Influence**	
Online Friend Network Size	.23(.08)***
Online Friend Network Tie Closeness	.54(.12)***

Note:

***p < .01,

**P < .05,

^†^p < .1.

Coefficients are standardized.

### Friend network

*Friend Network Size* related positively to *Friend Network Emotional Support* (b = .17, p < .01), *Friend Network Confidant Support* (b = .15, p < .01), *Friend Network Anti-Smoking Social Influence* (b = .16, p < .01) and *Friend Network Pro-Smoking Social Influence* (b = .18, p < .01). In addition, *Friend Network Tie Closeness* related positively to *Friend Network Emotional Support* (b = .36, p < .01), *Friend Network Confidant Support* (b = .37, p < .01), *Friend Network Anti-Smoking Social Influence* (b = .43, p < .01), and *Friend Network Pro-Smoking Social Influence* (b = .22, p < .01).

### Online friend network

*Online Friend Network Size* related positively to *Online Friend Network Emotional Support* (b = .16, p < .01), *Online Friend Network Confidant Support* (b = .17, p < .01), *Online Friend Network Anti-Smoking Social Influence* (b = .09, p < .10) and *Online Friend Network Pro-Smoking Social Influence* (b = .23, p < .01). Moreover, *Online Friend Network Tie Closeness* related positively to *Online Friend Network Emotional Support* (b = .73, p < .01), *Online Friend Network Confidant Support* (b = .61, p < .01), *Online Friend Network Anti-Smoking Social Influence* (b = .74, p < .01) and *Online Friend Network Pro-Smoking Social Influence* (b = .54, p < .01).

### 7-day abstinence

*Family Network Emotional Support* related marginally and negatively to *7-day Abstinence* (b = -.09, p = .062). *Family Network Confidant Support* related marginally and negatively to *7-day Abstinence* (b = -.08, p = .091). *Family Network Pro-Smoking Social Influence* related significantly and negatively to *7-day Abstinence* (b = -.23, p = .020). *Online Friend Network Emotional Support* related marginally and positively to *7-day Abstinence (b =* .*14*, *p =* .*069)*. None of the other relationships with *7-day Abstinence* were significant (see Table 3).

**Table 3:** Logistic Regression models regressing 7-day abstinence onto social support, social influence, demographics, and cigarettes per day smoked for family, friend, and online friend networks (n = 123).

	**Β(SE)**
Family Network Emotional Support	-.09(.05)†
Family Network Confidant Support	-.08(.05)†
Family Network Anti-Smoking Social Influence	-.07(.07)
Family Network Pro-Smoking Social Influence	-.23(.10)**
Friend Network Emotional Support	.01(.04)
Friend Network Confidant Support	.03(.04)
Friend Network Anti-Smoking Social Influence	-.10(.07)
Friend Network Pro-Smoking Social Influence	.07(.07)
Online Friend Network Emotional Support	.14(.08)†
Online Friend Network Confidant Support	.07(.07)
Online Friend Network Anti-Smoking Social Influence	-.16(.16)
Online Friend Network Pro-Smoking Social Influence	.17(.13)

Note:

***p < .01,

**P < .05,

^†^p < .10.

Coefficients are standardized.

## Discussion

Overall, our results indicated that for family, friend, and online friend network ties both network size and tie closeness related significantly and positively to nearly all of the social support and social influence constructs under study. Moreover, the relationships between tie closeness and both social support and social influence constructs were relatively stronger than those for network size for the friend network, and even more so for the online friend network. Our findings suggest that ties to friends, and especially online friends, may be conduits of social support and social influence. Our findings also indicate that support from online friends may be uniquely helpful in the quit attempt.

We also found that pro-smoking social influence from the family network, which was related to family network size but not tie closeness, related negatively to smoking abstinence. In addition, emotional support and confidant support from the family network, which were positively related to both family size and closeness, related marginally and negatively to smoking abstinence. These findings may indicate that family members have the potential to compromise adults’ quit smoking attempts, perhaps through exerting pro-smoking social influence (e.g., smoking themselves or accepting smoking), or by providing emotional or confidant support which may not support the quit attempt. These findings suggest new insights into instances when familial social support and social influence may be health compromising for the quit attempt among this adult sample.

In contrast, our results indicate that online friends may have potential to promote adults’ quit-smoking attempts. Though marginally significant, the only predictor that related positively to 7-day abstinence was emotional support from online friends. In addition, emotional support from the online friend network related positively to network size, but even more so to tie closeness. Taken together, these findings suggest that close online friends have the potential to be conduits of social support and social influence for adults trying to quit smoking and may facilitate quit attempts.

To our knowledge, the current study is the first to examine characteristics of three egocentric networks—family, friends and online friends—in relation to the social support and social influence these ties transmit, and how the characteristics of these network ties relate to adult smoking abstinence. Our elicitation of these three types of egocentric networks is a key measurement innovation relative to existing studies. Such studies have examined the roles of social support and social influence from friends and family in relation to quitting smoking among adults without parsing respondents’ perceptions of the number and closeness of their ties, the social support, and social influence perceived by the respondent as provided by each family member, friend and online friend network tie, measured egocentrically. Moreover, our study also considers the role of online friends, which is another innovation to the studies which have focused on in-person social ties. Moreover, prior studies have not examined the domains of social support and social influence examined herein, nor have such studies examined these relationships across the three types of egocentrically defined network ties we examined.

In addition, the social network ties we examined were naturally occurring, both in person and online, meaning that they were not formed within the context of a health behavior change intervention. Examining such ties is novel given the numerous existing studies of adults trying to quit smoking within the context of health behavior change interventions. Our findings indicate that naturally occurring online friend ties, especially close ties, may have the potential to be conduits of social influence and social support in the quit attempt, which is consistent with past research indicating that naturally occurring social network ties are strong conduits of both social support [30] and social influence [55].

The relationships between the social network characteristics of size and closeness and the social support and social influence constructs were nearly all in a positive direction, and nearly all significant, consistent with many past findings from studies of in-person egocentric networks. Perhaps having more or closer relationships in each type of network may lead to increased access to social support, or perhaps having more social support leads to forming more and closer ties.

Our study is also consistent with past research which indicated that friends are major sources of social support and social influence among adults [56]. In addition, the positive relationships we observed between the family network characteristics and social support and social influence constructs are consistent with past studies indicating that the family is an important social network for adults [41].

We also found that online friend network size and especially closeness related relatively more strongly in general than these constructs defined for the other networks to the social support and social influence constructs. These findings may suggest that close online friends may be strong conduits of social support and social influence. This is intriguing given that online ties operate in a decontextualized virtual space without the same cues and reinforcement of in-person relationships. Perhaps for adults, the maintenance of online friendships may be less burdensome than in-person friend or family ties. Regarding the two network characteristics under study, in general the closeness of ties related more strongly to the social support and social influence constructs than did network size and closeness of online friend ties was the most important. It is surprising that close online friend ties related to social support and the social influence outcomes more strongly than close friend or family ties. We are not aware of other studies of smoking cessation among adults which have examined close ties that formed naturally among online adult friends independent of an intervention, so little is known about how such ties function. What the construct of closeness to online friends encompasses also warrants further study, to understand its domains, as this kind of closeness may differ from the closeness of in-person friend or family ties. It may also differ from commonly accepted conceptualizations of tie strength, for instance, that of Granovetter (1973), which warrants future study.

Overall, our findings that both network size and tie closeness generally related positively to social support and social influence across the three networks are consistent with past studies [37, 57, 58]. However, we are not aware of past studies which have measured the two network characteristics of size and closeness, across the three types of networks under study including the online friend network, and measured associations with social support, social influence, and adult smoking abstinence. Consequently, the current study offers new insights into how these relationships play out across these three major types of adult social network ties.

Regarding predictors of smoking abstinence, anti-smoking social influence from each type of network tie was unrelated to smoking abstinence. Perhaps anti-smoking social influence is not well received or not perceived as constructive in the context of the social relationships under study. In contrast, family pro-smoking social influence, which related to having more but not closer family ties, related negatively to smoking abstinence. Likewise, family emotional and confidant support, both of which related to having more and closer family ties, were marginally and negatively related to abstinence. Hence, the family network’s social support and pro-smoking influence functions related to reduced quitting among adult smokers. Past literature suggests that people are influenced by friends to engage in risk behaviors [59], but our findings suggest that this influence may extend to family members. Moreover, there is a vast literature on adolescent smoking which documents the positive relationship between friends’ peer influences and smoking behavior [60], and our study indicates that adult smokers may experience pro-smoking influence from family.

Lastly, our findings indicate that one factor that may promote quitting smoking among adults is emotional support from the online friend network, perhaps based on its size and especially its closeness. Overall, rather than viewing friends as exerting pro-smoking influence, family members may do this among adults, but their online friends may have the potential to counter this by providing the emotional support that may promote abstinence.

### Implications

Our findings highlight the importance of future research which examines the social support functions of online friend network ties, which are independent of a health behavior change intervention. Our findings suggest that naturally occurring online friend ties may function similarly to family and friend ties, in terms of relating positively to perceptions of social support and social influence; but, regarding the online friend network, tie closeness relates even more strongly to perceptions of social support and social influence. Specifically, based on our findings, the closeness of online friend ties relates especially strongly to emotional and confidant social support and to pro- and anti-smoking social influence. In addition, emotional support from online friends relates marginally and positively to smoking abstinence. Thus, online friend ties warrant much more study to understand the social support and social influence functions of these ties, and how they relate to smoking abstinence. In addition, it is possible that the closeness of online friendship ties may be leveraged for the delivery of online smoking cessation interventions.

More generally, our findings highlight the importance of future research which examines the emotional and confidant support and pro-smoking and anti-smoking social influence functions of these three types of network ties, specifically close ties, and how there may be differences in the support and influence functions of these ties. A better understanding of how these ties function may provide insight into how to utilize these different networks in health behavior change interventions to reinforce the quit attempt and maintenance of abstinence behavior over time. Our findings also warrant further investigation of what the construct of closeness of online ties comprises.

### Limitations

The current study has a number of limitations. The adults in our sample were limited to frequent Facebook users given that this was an inclusion criterion of the randomized controlled trial our study data came from. Therefore, those in our sample were already frequent social media users, and thus may have been more likely than those who were not frequent social media users to form close online ties. Moreover, the sample was mostly female adults, therefore it is not clear that the results of the study would generalize to adult male populations, as it is possible that social support processes work differently across genders and in their relationship to smoking abstinence. However, this remains to be tested in future research. Regarding our abstinence measure, because it was self-reported, it is possible that some participants falsely reported their abstinence behavior which could have affected the distribution of this variable. Another limitation is that we did not collect information about the smoking status of participants’ family, friend and online friend network members and is left for future studies to examine the influence of specific network members. Lastly, this study only examined an adult sample, so it will be useful to understand how the study relationships play out in other populations such as adolescents, whose social worlds are inextricably linked to social media.

## Conclusion

Our study examined relationships between two theoretically salient social network characteristics that influence health, social network size and closeness, and key domains of social support and social influence, across three major types of social ties maintained by adults: family, friend, and online friend ties. We also examined whether social support and social influence related to abstinence from smoking. Overall, the closeness of ties to friends and especially online friends related most strongly to social support and social influence, with the effects for family being somewhat weaker though still generally significant. Interestingly, online friend ties mattered most for promoting smoking abstinence. Family members’ pro-smoking influence and emotional and confidant support were marginally and negatively related to abstinence. Overall, our findings suggest the importance of better understanding the social support and social influence functions of online friend ties as compared to in-person friend and family ties. Online friends are an elusive but potentially powerful set of individuals to identify and leverage to provide social support and social influence for adult smoking cessation.

## Supporting information

S1 Data (DTA)
